# Supplementary material for: Rate of glycemic control and associated factors among type two diabetes mellitus patients in Ethiopia: A cross sectional study
Source: PLoS One. 2021 May 11;16(5):e0251506. doi: 10.1371/journal.pone.0251506 (PMC8112661; doi:10.1371/journal.pone.0251506)
Supplement: S1 File — (DOCX) [file pone.0251506.s001.docx]

***Annex 1: Consent Form***

Greeting

My name is ______________________________. I am studying clinical pharmacy at the University of Gondar. I would like to ask you a few questions. The interview would take 5-10 minutes of your time. The purpose of this study is to assess poor glycemic control and associated factors in this hospital. This will be helpful for improving the glycemic level by identifying the factor and giving intervention. Your participation is completely voluntary. You can refuse to answer any questions and/or withdraw from the study at any time without a problem to you or the services you get in the hospital. All your responses will remain strictly confidential: the hospital staff will not have access to your responses. Your name will not be recorded and your responses will not be linked to your identity at any time. Do I have your permission to continue?

Yes No if yes, continue to the question. If No, skip to the next respondent

Date of Interview_________________ Name of data collector ______________ patient chart number ________

***Annex 2: Interview Questionnaire***

**Part I: socio demographic characters of participants**

1. Sex A. male B. female
2. How old are you (years) ………………
3. Where is your current Residence? A. Urban B Rural
4. What is your Educational level? A. unable to read and write B. able to read and write C. primary school D. secondary school E tertiary and above
5. What is your marital status? A. single B. Married C. Widowed D. Divorced
6. What is your main Occupation? A. student B. Employed C. Housewife D. Merchant E. others
7. What is your Religion? A. orthodox B. protestant C. Muslim D. others
8. What is your Ethnicity? A. Oromo B. Amhara C. Somali D. Tigrae E. other

**Part II: disease and drug related characteristics of study participant**

1. Do you have a family history of Diabetes mellitus? A. Yes B. No

2. How long is it since you found out that you have Diabetes mellitus? ……………….

3. For how long you have taken drug (years) …………………

**Part III: interaction with the pharmacist**

1. How do you rate your interaction with the Pharmacist? A. poor B. Moderate C. Good
2. When you taking your drug from the pharmacist in which language would you prefer to communicate? A. Amharic B. Afan Oromo C. Adarigna D. Somali E _________other(specify)

3. What is the clarity of the pharmacist advice about your drug? A. clear B. not clear

4. Are you satisfied with the overall pharmaceutical service you obtained during taking your drug from the pharmacist? A. Yes B. No

**Part IV. Knowledge, Attitude and Practice of T2DM patients**

**Knowledge of T2DM patients**

1. What is diabetes mellitus? A. correct answers B. incorrect answer
2. What type of DM you had? A. correct answers B. incorrect answer
3. What are the risk factors for T2DM? A. correct answer B. Incorrect answer
4. What are the complications of T2DM? A. correct answer B. incorrect answer
5. What is the effect of regular exercise on on T2DM? A.correct answer B.incorrect answer
6. What is the effect of extra sugar intake on T2DM ? A. correct answer B. incorrect answer
7. What is the effect of smoking on T2DM? A. correct answer B. incorrect answer
8. What types of drugs used for your DM? A. correct answer B. incorrect answer
9. What is the side effect of the drugs you took? A. correct answer B. incorrect answer
10. What to do when you miss your drug? A. correct answer B. incorrect answer
11. What to do when you become in hyperglycaemic crisis? A correct answer B. incorrect answer
12. What to do when you become hypoglycaemic? A. correct answer B. incorrect answer

**Attitude of T2DM patient**

1. Did you think controlling blood sugar is necessary for DM? A. yes B. no
2. Did you think regular exercise can help to control blood sugar? A. yes B. no
3. Did you think smoking causes poor blood sugar control? A. yes B. no
4. Did you think diet alone blood sugar control is better than medication with diet glycaemic control A. yes B. no
5. Did you think weight loss can prevent the complications diabetes mellitus? A. yes B. no
6. Did you think fruits and vegetables are better than starchy food? A. yes B. no
7. Did you think diabetic drugs can control the blood sugar level? A. yes B. no
8. Did you think diabetic drug have side effect on your body? A. yes B. no

**Practice of T2DM patient**

1. Did you keep a healthy eating plan? (avoide sugar, decrease fat, eat vegetables ....)

A. Yes B. No

1. Did you exercise regularly? A. Yes B. No

3. Did you smoke cigarette? A. Yes B. No

1. Did you drink alcohol? A. Yes B. No
2. Did you eat food on time? A. Yes B. No
3. Did you come on your appointment day regularly? A. Yes B. No
4. Did you add extra sugar on your regular diet? A Yes B. No
5. Did you keep your foot from trauma? A. Yes B. No
6. Did you use self-blood glucose monitoring? A. yes B. No
7. Did you take your drug on time? A. Yes B. No

***Annex 3: Chart review Questionnaire***

**Part I: Disease and drug related characteristics of study participant**

1. Body mass index ……………..kg/m

2. Did the patient have Comorbid diseases? A. Yes B. No

3. Did the patient have Diabetes complication? A. yes B. No

4. If the answer is yes in “Question 3” which of the following did the patient have?

A. Retinopathy B. Nephropathy C. Neuropathy D. Angina pectoris and Myocardial infraction E. Peripheral arterial disease E. Others

5. What type of treatment the patient take? A. OHD B. OHD + insulin C. Insulin

**Part II: selected drug for comorbidity**

| Co morbid condition | Selected drug |
| --- | --- |
|  |  |
|  |  |
|  |  |
|  |  |

**Part III: Laboratory investigation**

**Fasting blood sugar**

| Date/ Month/year |  |  |  |
| --- | --- | --- | --- |
| FBS |  |  |  |

**Blood Pressure**

| Date/ Month/year | |  |
| --- | --- | --- |
| BP | SBP |  |
|  | DBP |  |

**Lipid profile**

| Date/Month/year |  |
| --- | --- |
| LDL |  |

ቅፅ አንድ፡ ፍቃደኝነትን መጠየቂያ

ሰላም

ስሜ _______________________ይባላል. በጎንደር የኒቨርስቲ ክሊኒካል ፋርማሲ እያጠናሁ ነው. የተወሰኑ ጥያቄዎች ልጠየቀዎት አስቤለሁ. ጥያቄዎቹውም ከ 5 እስከ 10 ደቂቃ ከርሶዎ ጊዜ ይፈልጋሉ. የዚህ ጥናት ዋና አላማ በዚህ ሆስፒታል ውስጥ የስኳር ህመምቶኞችን የደም ዉስጥ የስኳር መጠን እና የስኳር መጠኑ እንዲጨምር የሚያደርጉትን ባህሪያት ያካትታል. ጥናቱ ሲጠናቀቅ የስኳር መጠኑ እንዲጨምር የሚያደርጉትን ባህሪያት ላይ ትምህርት ይሰጣል. የርሶዎ ተሳትፎ ሙሉ ለሙሉ በርሶዎ ፈቃደኝነት ላይ የተመሰረተ ነዉ. በማንኛዉም ሰአት ርሰዎን ከቃለ መጠየቁ ማግለል ይችላሉ. ሁሉም መልሰዎ በሚስጥር ይቀመጣሉ. ማንኛዉም የሆስፒታሉ ሰራተኛ የርሶዎን መልስ አያገኙትም. ፍቃደኛ ነዎት ጥያቄዎቼን ልቀጥል ?

አዎ አይደለሁም ¨መልሱ አዎ ከሆነ መቀጠል ካልሆነ ወደ ቀጣይ ተሳታፊ መሻገር.

የቃለ መጠይቁ ቀን _________________ የመረጃ ሰብሳቢዉ ሰም ______________

የታካሚዉ ካርድ ቁጥር ________

**የቃለ-መጠይቅ መጠይቅ**

1. ማህበራዊ ዳይሞግራፊክ
2. ፆታ. ሀ. ወንድ ለ. ሴት
3. እድሜዎ ስንት ነው?
4. አሁን ያለዎት መኖሪያ የት ነው? ሀ. ከተማ ለ. ገጠር
5. የትምህርት ደረጃዎ ምንድን ነው? ሀ. ማንበብና መፃፍ አለመቻል ለ. ማንበብና መፃፍ መቻል ሐ. የመጀመሪያ ትምህርት ቤት መ. ሁለተኛ ደረጃ ትምህርት ቤት ሠ. የከፍተኛ ሁለተኛ ደረጃ ትምህርት ቤት እና ከዚያ በላይ
6. የጋብቻ ሁኔታወ ምንድን ነው? ሀ. ያለገባ/ች ለ. ያገባ/ች ሐ. የሞተበት/ባት መ. የፈታ/ች
7. የእርስዎ ዋና ስራ ምንድን ነው? ሀ. ተማሪ ለ. ተቀጥሮ የሚሰራ ሐ. የቤት እመቤት መ. ነጋዴ ሠ. ሌላ
8. ሀይማኖትዎ ምንድን ነው? ሀ. ኦርቶዶክስ ለ. ጴንጤ ሐ. ሙስሊም መ. ሌላ
9. የእርስዎ ብሄር ምንድን ነው? ሀ. ኦሮሞ ለ. አማራ ሐ. ሶማሌ መ. ትግሬ ሠ. ሌላ
10. ከበሽታና ከመድሀኒት ጋር የተያያዙ የጥናቱ ተሳታፊዎች ባህሪያት
11. እርስዎ የስኳር ህመምተኞች የቤተሰብ ታሪክ አለዎት ሀ. አዎ ለ. የለኝም
12. የስኳር ህመም እንዳልዎት ካወቁ ወዲህ ምን ያህል ጊዜ ነው?
13. ለምን ያህል ጊዜ መድሀኒት ወስደዋል?
14. እርስዎ የሚወሰዷቸው የስኳር መድሀኒቶች ጠቅላላ ብዛት ምን ያክል ነው? ሀ. አንድ ለ. ሁለት ሐ. ሶስት ወይም ከዚያ በላይ
15. ከመድሃኒት ባለሙያው ጋር ያለወን ግንኙነት
16. ከመድሃኒት ባለሙያ ጋር ያልዎትን ግንኙነት እንዴት ይገመግሙታል? ሀ. ደካማ ለ. መካከለኛ ሐ. ጥሩ
17. መድኃኒት በሚወስዱበት ወቅት በየትኛው ቋንቋ መነጋገር ይመርጣሉ

ሀ. አማረኛ ለ. ኦሮምኛ ሐ. አዳርኛ መ. ሶማልኛ መ. ሌላ

1. ስለመድሀኒትዎ የመድሀኒት ባልሙያ የሚሰጥዎት የምክር አገልግሎት እንዴት ነው? ሀ. ግልፅ ነው ለ. ግልፅ አይደለም
2. በክትትልዎ ወቅት ከመድሃኒት ባለሙያው ባገኙት ጠቅላላ የመድሃኒት አገልግሎት ደስተኛ ነዎት? ሀ.አዎ ለ. አደለሁም
3. **የስኳር ህመምቶኞች** ዕውቀት፣ አመለካከት እና ተግባር

**የስኳር ህመምቶኞች እውቀት**

1. የስኳር በሽታ ምንድን ነው? ሀ. በትክክል መልሰዋል ለ. በትክክል አልመለሱም
2. ምን አይነት የስኳር በሽታ አለበዎት? ሀ. በትክክል መልሰዋል ለ. በትክክል አልመለሱም
3. ለስኳር በሽታ መንሰኤዎች ምንድን ናቸው? ሀ. በትክክል መልሰዋል ለ. በትክክል አልመለሱም
4. የስኳር በሽታ ምን አይነት በሽታዎችን ያመጣል? ሀ. በትክክል መልሰዋል ለ. በትክክል አልመለሱም
5. ስፓርት መስራት ለስኳር በሽታ ምን ጥቅም አለው? ሀ. በትክክል መልሰዋል ለ. በትክክል አልመለሱም
6. ተጨማሪ ስኳር መዉሰድ ለስኳር በሽታ ምን ጉዳት አለው? ሀ. በትክክል መልሰዋል ለ. በትክክል አልመለሱም
7. ሲጋራ ማጭስ ለስኳር ህመም ምን ችግር አለው? ሀ. በትክክል መልሰዋል ለ. በትክክል አልመለሱም
8. ምን አይነት የስኳር መድሀኒት ይጠቀማሉ? ሀ. በትክክል መልሰዋል ለ. በትክክል አልመለሱም
9. የሚውስዱት መድሀኒት ምን አይነት የጎንዩሽ ጉዳት ያመጣል? ሀ. በትክክል መልሰዋል ለ. በትክክል አልመለሱም
10. መድሃኒትዎን ቢረሱ ምን ያደረጋሉ? ሀ. በትክክል መልሰዋል ለ. በትክክል አልመለሱም
11. በደመዎ ውስጥ ያለው የስኳር መጠን በጣም ቢጨምር ምን ያደረጋሉ? ሀ. በትክክል መልሰዋል ለ. በትክክል አልመለሱም
12. በደመዎ ውስጥ ያለው የስኳር መጠን በጣም ቢቀነስ ምን ያደረጋሉ? ሀ. በትክክል መልሰዋል ለ. በትክክል አልመለሱም

**የስኳር ህመምተኞች አመለካከት**

1. በደመዎ ውስጥ ያለው የስኳር መጠን መቆጣጠር ለስኳር በሽታ ጠቃሚ ነው ብለው ያስባሉ? ሀ. አዎ ለ. አላስብም
2. በቋሚነት ስፓርት መስራት በደመዎ ውስጥ ያለውን የስኳር መጠን መቆጣጠር ይጠቅማል ብለው ያስባሉ? ሀ. አዎ ለ. አላስብም
3. ሲጋራ ማጭስ በደመዎ ውስጥ ያለውን የስኳር መጠን ከቁጥጥር ወጭ ያደረገዋል ብለው ያስባሉ? ሀ. አዎ ለ. አላስብም
4. የአመጋገብ ሆኔታዎን ብቻ ማስተካከል በደመዎ ውስጥ ያለውን የስኳር ‹መጠን ለመቆጣጠር ከአመጋገብዎ ሁኔታ ጋር አስተካከለው መድሃኒት ከመውሰድ በተሸለ ሁኔታ የስኳር መጠንዎን የቆጣጠርዋል ብለው ያስባሉ?

ሀ. አዎ ለ. አላስብም

1. የክብደት መጠንዎን መቀነስ ከስኳር ጋር ተያይዘው የሚመጡ በሽታዎች ይከላከላል ብለው ያስባሉ? ሀ. አዎ ለ. አላስብም
2. ስኳርነት ያላቸው ምግቦች ከመጠቀም ይለቅ ፍራፍሬና አትክልቶችን መጠቀም የተሸለ ነው ብለው ያስባሉ? ሀ. አዎ ለ. አላስብም
3. የስኳር መድሃኒቶች በደመዎ ውስጥ ያለውን የስኳር መጠን ይቆጣጠረዋል ብለው ያስባሉ? ሀ. አዎ ለ. አላስብም
4. የስኳር መድሃኒቶች ለሰውነትዎ የጎንዩሽ ጉዳት ያመጣሉ ብለው ያስባሉ? ሀ. አዎ ለ. አላስብም

**የስኳር ህመምተኞች ተግባር**

1. ጤናማ የምግብ እቅድ ያስቀምጣሉ? (ለምሳሌ አትክልት ፍራፍሬ የመሳሰሉትን መጠቀም)? ሀ. አዎ ለ. አላስቀምጠም
2. ዘውትር ስፓርት ይሰራሉ? ሀ. አዎ ለ. አልሰራም
3. ሲጋራ ያጨሳሉ? ሀ. አዎ ለ. አላጨስም
4. አልኮል ይጠጣሉ? ሀ. አዎ ለ. አልጠጣም
5. ምግብዎን ሰዓቱን ጠብቀው ይመገባሉ? ሀ. አዎ ለ. አልመገብም
6. የቀጠሮ ጊዜዎን ጠብቀው ይመጣሉ? ሀ. አዎ ለ. አልምጣም
7. ከእለታዊ ምግብዎ ላይ ተጨማሪ ስኳር ይጨምሮበታል? ሀ. አዎ ለ. አለጨምርም
8. እግሮዎን ከተለያዩ እንቅፋቶች ይጠብቃሉ? ሀ. አዎ ለ. አልጠብቅም
9. በግልዎ የደመዎን የሥኳር መጠን መለኪያ ይጠቀማሉ ? ሀ. አዎ ለ. አልጠብቅም
10. መድሃኒትዎን ሰዓቱን ጠብቀው ይወሰዳሉ? ሀ. አዎ ለ. አልውስድም

ለትብብርዎ አመሰግናለሁ !!!
