# Supplementary material for: Rate of glycemic control and associated factors among type two diabetes mellitus patients in Ethiopia: A cross sectional study
Source: PLoS One. 2021 May 11;16(5):e0251506. doi: 10.1371/journal.pone.0251506 (PMC8112661; doi:10.1371/journal.pone.0251506)
Supplement: S2 File — (DOCX) [file pone.0251506.s002.docx]

***Pretest Result***

**Socio demographic characteristics of T2DM patients**

In this pretest study, a total of 22 patients were participated. More than half of participants 12(54.5%) were females. From those participants, 14(63.6%) belong in the age group of 40-59 years. Thirteen (59.1%) participants live in urban. The majority of participants 18(81.8%) were married. Seven participants (31.8 %) were employed (table 1)

Table 1: Socio demographic characteristics of 22 T2DM patients who were attending Dilchora Referral Hospital, April 2019.

| **Characteristics** | | **Frequency**  **(%)** | **Glycemic level** | |
| --- | --- | --- | --- | --- |
|  |  |  | **Good**  **(%)** | **Poor**  **(%)** |
| Sex | Female  Male | 12(54.5)  10(45.5) | 2(16.7)  8(80) | 10(83.3)  2(20) |
| Age (years) | 18-39  40-59  > 60 | 14(13.6)  5(22.7)  3(66.7) | 4(28.6)  4(80)  2(33.3) | 10(71.4)  1(20)  1(40) |
| Current residence | Urban  Rural | 13(59.1)  9(40.9) | 4(30.8)  6(67.7) | 9(69.2)  3(33.3) |
| Educational level | Unable to read and write  Able to read and write  Primary school  Secondary school  Tertiary and above | 2(9.1)  2(9.1)  9(40.9)  3(13.6)  6(27.3) | 0  1  3(33.3)  2  4(66.7) | 2  1  6(66.7)  1  2(33.3) |
| Marital status | Single  Married  Divorced | 3(13.6)  18(81.8)  1(4.5) | 3  6(33.3)  1 | 0  12(67.3)  0 |
| Occupation | Student  Employed  Housewife  Merchant  Daily laborer | 4(18.2)  7(31.8)  3(13.6)  2(9.1)  6(27.3) | 3  3(42.9)  0  0  4(67.3) | 1  4(57.1)  3  2  2(33.7) |
| Religion | Orthodox  Protestant  Muslim | 12(54.5)  1(4.5)  9(40.9) | 4(33.3)  1  5(55.6) | 8(66.7)  0  4(44.4) |
| Ethnicity | Oromo  Amhara  Somali  Tigrae | 8(45.5)  10(36.4)  3 (13.6)  1(4.5) | 5(62.5)  4(40)  0  1 | 3(37.5)  6(60)  3  0 |

**Disease and drug related characteristics of T2DM patients**

More than half of participants 18(81.8%) had no family history of DM. The majority of the study participants 17(77.3%) were living with DM for seven years or more. Nearly to fifty-five percent of participants 12(54.5%) had comorbid disease. About 13(59.1%) participants had diabetic complications. More than half of the study participants 16(72.7%) took a diabetic drug for seven or more years and Out of the total participants, 11(50%) participants were taking insulin alone (table 2 )

Table 2: disease and drug related characteristics of T2DM patients who were attending Dilchora Referral Hospital, April 2019.

| **Characteristics** | | **Frequency (%)** | **Glycemic level** | |
| --- | --- | --- | --- | --- |
|  |  |  | **Good (%)** | **Poor**  **(%)** |
| Family history of DM | Yes  No | 4(18.2)  18(81.8) | 1  9(50) | 3  9(50) |
| Duration of DM | < 7 years  > 7 years | 5(22.7)  17(77.3) | 2  8(47.1) | 3  9(52.9) |
| Comorbid disease | Yes  No | 12(54.5)  10(45.5) | 5(41.7)  5(50) | 7(58.3)  5(50) |
| Diabetic complication | Yes  No | 13(59.1)  9(40.9) | 5(38.5)  5(55.6) | 8(61.5)  4(44.4) |
| Drug given for dyslipidemia | Atorvastatin | 3 | 1 | 2 |
| Drug given for hypertension | Enalapril  Enalapril + HCT  HCT  Nifidipine | 6  2  1  2 | 4  1  1  0 | 2  1  0  2 |
| Duration of DM treatment | < 7 years  > 7 years | 6(27.3)  16(72.7) | 3  7(43.8) | 3  9(56.2) |
| Types of treatment | Oral anti diabetic drug  Insulin  Oral anti diabetics + insulin | 6(27.3)  11(50)  5(22.7) | 2  6(54.5)  2 | 4  5(45.5)  3 |

*hearing loss & diarrhea MI=myocardial infraction

**Interaction of T2DM patients with pharmacist**

About 14(63.6%) participants had good interaction with the pharmacists. More than three fourth of participants 18(81.8%) prefer Amharic language to communicate with a pharmacist. Nearly to fifty percent of participants 10(45.5%) were not satisfied with the overall pharmaceutical service obtained from the pharmacists (table 3).

Table 3: Interaction of T2DM patients who were attending Dilchora Referral Hospital with pharmacist, April 2019

| ***Characteristics*** | | **Frequency**  **(%)** | **Glycemic level** | |
| --- | --- | --- | --- | --- |
|  |  |  | **Good**  **(%)** | **Poor**  **(%)** |
| rate of interaction with the pharmacist | Good  Moderate  Poor | 14(63.6)  4(18.2)  4(18.2) | 5 (35.7)  1  3 | 9 (64.3)  3  1 |
| Patient language preference to communicate with pharmacist | Amharic  Afan oromo  Somali  Woleytgna | 18(81.8)  3  2  3 | 6(33.3)  3  2  2 | 12(66.7)  0  0  1 |
| clarity of the pharmacist advice about their drug | clear  not clear | 14(63.6)  8(36.4) | 6(42.9)  3 | 8(57.1)  5 |
| Satisfied with overall pharmaceutical service get from pharmacists | Yes  No | 12(54.5)  10(45.5) | 4(33.3)  5(50) | 8(66.7)  5(50) |

**Clinical characteristics of T2DM patients**

Of the study participants, 10(45.5%) had overweight body mass index. Thirteen participants had a systolic blood pressure level of less 140mmHg. Ten participants had LDL laboratory results (table 4)

Table 4: Clinical characteristics of T2DM patients who were attending Dilchora Referral Hospital, April 2019

| **Clinical characteristics** | | **Frequency**  **(%)** | **Glycemic Level** | |
| --- | --- | --- | --- | --- |
|  |  |  | **Good**  **(%)** | **Poor**  **(%)** |
| Body mass index | Underweight  Normal  Overweight  Obese | 1(4.5)  8(36.4)  10(45.5)  3(13.6) | 1  5  2  1 | 0  3  8  2 |
| Blood pressure level(mmHg) | DBP > 90  DBP < 90  SBP >140  SBP <140 | 2(9.1)  20(90.9)  13(59.1)  9(40.9) | 9(50)  207(55.1)  50(52.1)  166(55.7) | 9(50)  169(44.9)  46(47.9)  132(44.3) |
| Low density lipoprotein level(mg/dl) | >100  < 100 | 6  4 | 3  0 | 3  4 |

**Knowledge, Attitude and practice of T2DM patients**

More than two third of respondents 16(72.7) had inadequate knowledge about T2DM. seventeen participants had a good attitude towards T2DM. More than half of the participants (59.1%) had poor practice of DM (table 5).

Table 5: Knowledge, Attitude and practice of T2DM patients who were attending in Dilchora Referral Hospital, April 2019

| **Characteristics** | | **Frequency**  **(%)** | **Glycemic level** | |
| --- | --- | --- | --- | --- |
|  |  |  | **Good**  **(%)** | **Poor**  **(%)** |
| Level of Knowledge | Adequate knowledge  Inadequate knowledge | 6(27.3)  16(72.7) | 3  6(37.5) | 3  10(62.5) |
| Level of Attitude | Good attitude  Poor attitude | 17(77.3)  5(22.7) | 8(47.1)  1 | 9(52.9)  4 |
| Level of Practice | Good practice  Poor practice | 9(40.9)  13(59.1) | 4  5(38.5) | 5  8(61.5) |
